# Thermo-Mechanical Properties of Cis-1,4-Polyisoprene: Influence of Temperature and Strain Rate on Mechanical Properties by Molecular Dynamic Simulations

**DOI:** 10.3390/polym17091179

**Published:** 2025-04-26

**Authors:** Tannaz Alamfard, Cornelia Breitkopf

**Affiliations:** Institute of Power Engineering, Faculty of Mechanical Science and Engineering, Technical University Dresden, 01069 Dresden, Germany; cornelia.breitkopf@tu-dresden.de

**Keywords:** Green-Kubo method, thermal conductivity, glass transition temperature, equilibrium molecular dynamic simulation (EMD), autocorrelation function, cis-1,4-polyisoprene, stress–strain behavior, elasticity modulus, uniaxial tensile deformation

## Abstract

Cis-1,4-polyisoprene is a widely used elastomer that demonstrates particular thermal and mechanical characteristics, in which the latter is influenced by temperature and strain rate. Molecular dynamic simulations were used to obtain thermal conductivities, glass transition temperatures (*T*_g_), and tensile deformation. Thermal conductivities were calculated by applying the Green–Kubo method, and a decrease in thermal conductivity was observed with increasing temperature. Density–temperature relations were used to calculate *T*_g_, which indicates the transition from the glassy to the rubbery state of the material, and this temperature influences mechanical properties. Investigation of the mechanical properties under uniaxial tensile deformation for constant strain rates indicates an increase in the stiffness and strength of the material at lower temperatures, while increasing molecular mobility at higher temperatures results in reducing these properties. The influence of strain rates at constant temperature highlighted the viscoelastic nature of the structure; increasing strain rates resulted in increases in stiffness, strength, elongation at maximum strength, and elongation at break because of restricted molecular relaxation time. The united-atom force field contributes to higher computational efficiency, which is suitable for large-scale simulations. These results provide important information on the thermo-mechanical properties and tunability of cis-1,4-polyisoprene, which supports applications in the production of interactive fiber rubber composites.

## 1. Introduction

Cis-1,4-polyisoprene, often known as NR or CPI, is a well-known elastomer due to its extensive use in the automotive and industrial sectors. CPI is an amorphous material that has been verified to crystallize during cooling as well as stretching [1]. The microscopic nature of heat conduction and the calculation of thermal conductivity can both be accomplished with molecular dynamics (MD) simulations. The two most popular methods are nonequilibrium molecular dynamics (NEMD) and equilibrium molecular dynamics (EMD) simulations. Fourier’s law of heat conduction [1] is used in the NEMD method to evaluate thermal conductivity. This involves applying a heat flux to a related structure and analyzing the temperature gradient that results; alternatively, it can be employed to determine a heat flux from an applied temperature gradient. The EMD method is predicated based on the linear response theory-derived Green-Kubo (GK) technique [2].

In both methods, temperature variations, which are approximately larger than 10K, produce nonlinear temperature profiles and unrealistically high heat fluxes. Moreover, since the molecular structure needs to be sufficiently large to accommodate long-wavelength and long mean-free-path (MFP) phonons, size effects might also exist for solids [3]. The molecular structure is in an equilibration state throughout the simulation with EMD. For solids, since EMD is less dependent on size, it is sometimes favored over NEMD. The size of the molecular structure should be sufficiently massive to contain a variety of wavelengths, but not necessarily the MFPs [1,4,5], because phonons are able to move past the supercell’s face and re-enter through opposite sides without any dispersion in periodic boundary conditions (PBCs).

A vital feature of polymers that affects their use in various fields, particularly automotive, aerospace, and electronics, is their thermal conductivity [6]. Accordingly, a lot of research has been performed in the last few decades to look into the structure–property connections for polymers, such as the impact of molecular weight, chain interactions, or domain sizes, in an effort to improve their thermal conductivity and explore novel application areas. Several studies have been conducted on these features of polymers, and quantitative comparisons of the modeling techniques used are also available [7,8,9].

Optimizing the efficiency of polymers in different industries requires an ability to calculate their thermal conductivity [6]. For instance, cis-1,4-polyisoprene, the main component of natural rubber, is utilized extensively in the manufacturing of a variety of rubber supplies, including tires, conveyor belts, sealing rings, and more [10]. These functions emphasize how crucial it is to comprehend and optimize polyisoprene’s thermal characteristics for industrial applications.

The low thermal conduction capability of NR substances leads to a number of significant issues. Low heat conduction of NR substances, for instance, is the cause of tire failures that lead to tire explosions. To obtain a better understanding of the heat conduction of rubber composites, several theoretical and experimental investigations have been conducted [11,12,13,14]. The thermal conductivity of NR composites is not much improved by these investigations, which appear to address the same issues as extremely high thermal conductive reinforcing agents. A microscopic analysis of the polymeric structure is required to describe the heat conduction in NR materials. Molecular dynamics (MD) simulation represents a knowledge of physical characteristics at the atomic level and may forecast attributes in a cost-effective and time-efficient manner. The accuracy of calculations [15,16] and the complexity [5,17,18,19,20,21,22] of the materials may be significantly improved by MD simulations due to recent advancements in computer technology and possible functionalities. A number of MD simulation investigations have been conducted to calculate the heat conductivity of polymers [5,19,20,21,22].

Another significant thermal characteristic of polymers is the glass transition temperature (*T*_g_), which represents a specific temperature at which an amorphous polymer shifts from a hard, glassy state to a soft, rubbery state [23]. The strength, flexibility, and durability of polymers are all impacted by the related glass transition temperature, which is crucial in defining their mechanical characteristics and uses [24]. The glass transition temperature, *T*_g_, is considered a significant factor in a lot of complex practical materials [25,26,27,28,29]. For instance, understanding the glass transition temperature is particularly important for materials such as poly(3,4-ethylenedioxythiophene) (PEDOT) [30], as it influences the crystallization and orientation of the material [31]. When the deposition temperature exceeds the *T*_g_ of approximately 100 °C, the crystallites in PEDOT undergo a reorientation process that minimizes interfacial energy, leading to a highly preferential face-on orientation. This enhanced crystallinity significantly impacts electrical conductivity and carrier mobility, making PEDOT increasingly valuable in advanced applications, including thermoelectric devices [31].

The characteristics of polymer chains, the surroundings of polymer chains, the properties of nanoparticles or solvents, and other factors have an influence on the glass transition temperature of a polymeric structure [32,33,34,35]. In order to prevent thermal destruction of material under adverse conditions, there is a need to produce materials with specific glass transition temperatures in other industries, such as solar technology and aerospace [36,37]. Therefore, in addition to empirical studies and theoretical advancements [38,39,40,41], computer simulations offer essential molecular-level knowledge that facilitates comprehension of polymer glass transition [42,43,44,45,46].

Neat polymers are crucial in many different types of technological applications; nonetheless, their relatively low strength and stiffness provide an apparent limitation. In order to increase the range of applications for these polymers, different reinforcements are employed to improve their mechanical and physical properties [47,48,49,50]. Since polymers have various molecular structures or composites, they represent a vast variety of mechanical properties, such as elasticity modulus. For example, the elasticity modulus of an elastic polymer, like low-density polyethylene (LDPE), is 0.1 GPa, which is low. On the other hand, the elasticity modulus of a stiff polymer, like polyether ether ketone (PEEK), can reach a high value of 24 GPa [51]. A number of different variables, including crystallinity, the arrangement of chains, and the existence of plasticizers or additives, have an influence on the changes in elasticity modulus [52].

Molecular simulations and computational modeling are crucial methods for examining the mechanical characteristics of materials in addition to experimental investigations [53,54,55,56]. Simulations offer important insights into the mechanical properties, stress resistance, and deformation of materials under different circumstances [57]. A more thorough knowledge of the fundamental processes governing the mechanical characteristics of polymeric materials at the atomic scale may be attained, especially through the use of atomistic simulations [58,59,60,61].

In the current study, the equilibrium molecular dynamic (EMD) simulation was used to examine the thermal conductivities of cis-1,4-polyisoprene as a function of the heat flux autocorrelation function. Thermal conductivities have been determined by means of the Green–Kubo technique. Simulations were conducted employing the Moltemplate software (version 2.20.3, Andrew Jewett, Los Angeles, CA, USA) by using the united-atom force field (OPLS-UA) and all-atom force field (OPLS-AA). Density–temperature correlations were additionally utilized to determine *T*_g_, which is the related temperature that affects mechanical characteristics of the material. Additionally, it was examined how temperature and strain rate influence the material’s mechanical characteristics, such as its elasticity modulus, maximum strength, elongation at maximum strength, and elongation at break.

## 2. Theoretical Formulation and Methods

### 2.1. Green–Kubo Method and Force Field Representation

The equilibrium molecular dynamics EMD technique can be employed with the fluctuation–dissipation theory to determine thermal conductivity [62]. Here, the time history of the equilibrium variations of the volume-averaged heat flux is applied to calculate the linear response of the system to a slight thermal disturbance. The thermal conductivity, *k*, is obtained using the Green–Kubo (GK) expression as follows [63,64]:

(1) $$k=\frac{V}{k_{B}T^{2}}\int_{0}^{\infty }\left\langle J_{x}\left(t\right)J_{x}\left(0\right)\right\rangle dt$$

in which $J_{x}$ denotes the heat flux in the *x* direction, $\left\langle J_{x}\left(t\right)J_{x}\left(0\right)\right\rangle$ represents the heat flux autocorrelation function (HFACF), *V* is the system volume, *T* the system temperature, and $k_{B}$ the Boltzmann constant.

The thermal conductivity of an isotropic system is typically determined by evaluating the average of the thermal conductivities along three directions (*x*, *y*, and *z*). By adding a factor of three to the denominator of the Green–Kubo formula, the average thermal conductivities along the *x*, *y*, and *z* axes are thus determined in the current study.

(2) $$k=\frac{V}{{3\;k}_{B}T^{2}}\int_{0}^{t_{c}}{\left\langle J\left(t\right)\cdot J\left(0\right)\right\rangle }_{t_{s}}dt$$

where $t_{s}$ represents the timeframe over which the ensemble average for calculating the heat flux autocorrelation function (HFACF) is accumulated. *J* denotes the heat flux vector, and $t_{c}$ is the finite correlation time over which the integration is performed. Two techniques have been developed for determining the heat flux vector [65]. A detailed discussion of the most common formula for calculating the heat flow vector with all related formulations can be found in [66]. The system energy in the non-polarizable OPLS force field is determined by adding up all of the intra-molecular and inter-molecular interactions [67,68,69,70]. The intra-molecular terms consist of bond stretching, angle bending, and torsional energies. The inter-molecular potential consists of Coulomb interactions and van der Waals forces, represented by 12-6 Lennard-Jones terms, which were entirely described in [66].

### 2.2. Glass Transition Temperature

The glass transition temperature (*T*_g_) of amorphous polymers is one of the most crucial thermal characteristics. A polymer changes from a rubber to a glass (an amorphous solid locked in a non-equilibrium state) as it cools through *T*_g_. Glass transition temperature is an inherent molecular structural characteristic that is controlled by local chain dynamics. Predictive simulations are a useful tool since the origin of glass transition is not entirely understood, even though material engineers currently determine *T*_g_ empirically using the dilatometric approach [71].

It is a typical procedure to use a thermostat to regulate the temperature and to create isobaric conditions in order to cool and/or heat up a model system at a steady rate in order to calculate *T*_g_ using MD simulations. Typically, the cool-down or heat-up is carried out in a stepwise manner over a specific amount of time, applying defined temperature decreases or increases. Understanding the glass transition depends on the thermal history of the structure, and heating/cooling rates have substantial impacts on its value [72]. Therefore, the system is subjected to the *NPT* ensemble at different temperatures, and the density values of the system are determined at each temperature separately. A consistent rise in density will result from decreasing the temperature. The density line that interpolates the density data has a distinct gradient shift at the *T*_g_ temperature [73].

By examining the linear change in density with respect to temperature in both the glassy and rubbery regimes, which have different slopes, the glass transition temperature (*T*_g_) is found. The temperature at which the density slope shifts from the rubbery to the glassy value is known as *T*_g_. In fact, two linear regression models are fitted to the low- and high-temperature regions, and *T*_g_ is determined by the temperature at which these regression lines intersect [74].

### 2.3. Uniaxial Tensile Simulation

A crucial mechanical characteristic that measures the stiffness of a material is its elasticity modulus, also known as its modulus of elasticity. This property is crucial for studying the mechanical behavior of materials and is defined as the ratio of stress to strain in the linear elasticity domain of a uniaxial deformation [75]. The elasticity modulus of polymers offers important information on their stiffness and elastic properties under applied stress, which is essential for a variety of applications [53].

EMD simulations of uniaxial deformation are used to describe the mechanical response of the cis-1,4-polyisoprene sample. In these simulations, the length of the simulation cell is continually expanded along one of its cubic directions at each MD step, while ambient pressure is maintained in the transverse directions using a barostat. The periodic boundary condition has been applied for three directions during MD simulation. The stress–strain curve is derived from uniaxial deformations carried out in the *x* direction.

### 2.4. Set-Up of Simulation and Creation of Model

**Figure 1:**
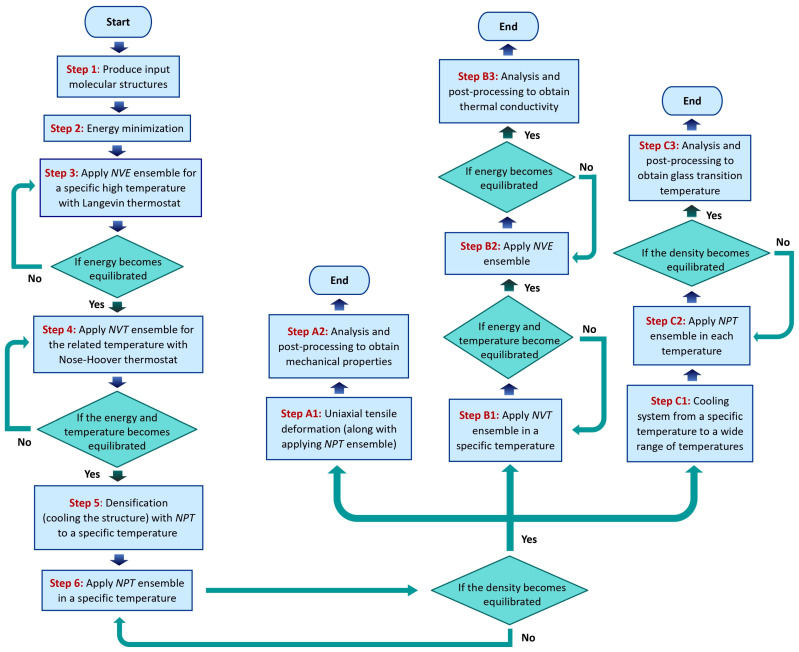
Flowchart that demonstrates modeling process and ensemble application.

The primary cis-1,4-polyisoprene chain model system was developed and replicated in a cubic cell in step 1 of Figure 1. Step 2 involved using the “minimize” LAMMPS command to minimize the energy of the system. In step 3, the simulation was conducted using Langevin dynamics at a high temperature using the *NVE* ensemble. This was followed by relaxation using an *NVT* ensemble at the same high temperature using a Nose–Hoover thermostat [10].

To obtain a more realistic and desired density for the simulated polymeric material, densification is carried out in step 5 using the *NPT* ensemble. Due to the arrangement of atoms and molecules, the system might be in a high-energy condition after densification. Therefore, in step 6, obtaining equilibration with the *NPT* ensemble provides a context to optimize the molecular framework by modifying bond lengths, bond angles, and dihedral angles in order to minimize potential energy and provide a configuration that is chemically practical. The equilibration will stabilize the system and provide a more realistic initial structure for the subsequent steps.

This initial configuration must be employed in three distinct stages (steps A, B, and C) in order to achieve mechanical characteristics, thermal conductivity, and glass transition temperature. In step A1, the uniaxial tensile deformation alongside the *NPT* ensemble will be applied to study mechanical characteristics of the polymeric structure. Step B1 will stabilize the temperature by applying the *NVT* ensemble at the relevant temperature in order to achieve thermal conductivity. If the temperature stabilizes, the *NVE* ensemble at the corresponding temperature would be applied in step B2 to determine the thermal behavior of the framework. In step C1, the structure will be cooled from a specified temperature to a wide range of temperatures in order to achieve density at various temperatures, which will lead to obtaining the glass transition temperature. In step C2, the structure would then be equilibrated with the *NPT* ensemble in each of these broad temperature ranges. The density, energy, and radial distribution function (RDF) of the material were investigated to obtain the equilibration state for the polymeric structure.

## 3. Results and Discussion

### 3.1. Representation of Cis-1,4-Polyisoprene in MD Simulations

**Figure 2:**
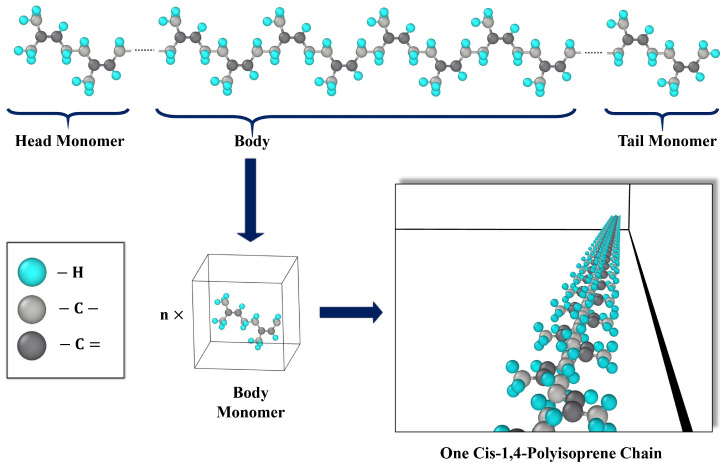
Creating cis-1,4-polyisoprene chain procedure with all-atom model.

In order to examine the effects of applying both all-atom and united-atom force fields on the mechanical characteristics, two models were developed for this study: the all-atom model and the united-atom model. As the molecular model for polymers with a united-atom force field was studied in [66,76], the molecular model of cis-1,4-polyisoprene with an all-atom force field was investigated in this section. The united-atom model is often used to describe hydrocarbons such as alkanes and alkenes and is very helpful for studying long-chain compounds [77]. Additionally, the united-atom model was taken into consideration to derive thermo-mechanical characteristics because it provided this possibility to apply a larger system size, a longer time scale, and reduced computational cost. In this study, the polymeric structure of cis-1,4-polyisoprene has been considered. The Moltemplate program (version 2.20.3, Andrew Jewett, Los Angeles, CA, USA) [78] produced the molecular model of the chain for both all-atom and united-atom force fields. The molecular models of the head group, repetition group (the chain’s body), and tail group [76] were created for the cis-1,4-polyisoprene chain in Figure 2.

**Figure 3:**
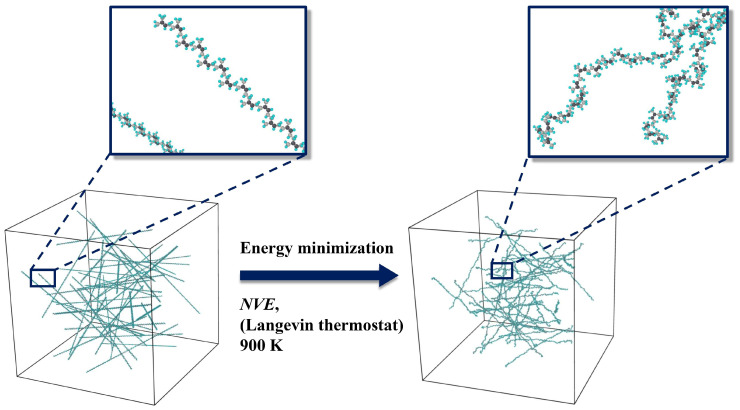
The polymeric structure after replication, applying energy minimization and NVE ensemble.

Figure 3 illustrates the random arrangement of a certain number of polyisoprene chains (depending on the objective of the related research) within a simulation cell applying Packmol software (version 20.3.5, Leandro Martínez, Campinas, Brazil) [79]. A tolerance factor of 2.0 Å was taken into consideration in Packmol. Figure 3 also displays the polymeric structure after applying energy minimization and the NVE Langevin thermostat at 900 K (steps 2 and 3 of Figure 1).

All simulations were conducted using the LAMMPS software (version 3 March 2020, Sandia National Laboratories, Albuquerque, NM, USA) [80]. The cutoff distance, van der Waals interactions (using a mixed geometric equation), and “special_bonds” coefficients settings are based on Ref. [66]. An overview of the Lennard–Jones potential parameters and other all-atom force fields utilized in the MD simulations is given in Table 1. These all-atom force field (OPLS-AA) parameters were obtained using the Moltemplate software [78] and applied to bond stretching, van der Waals, dihedral, and angle interactions. Due to the lack of some all-atom force field parameters for these combinations, a part of the dihedral and angle interactions were not included in the present study.

**Figure 4:**
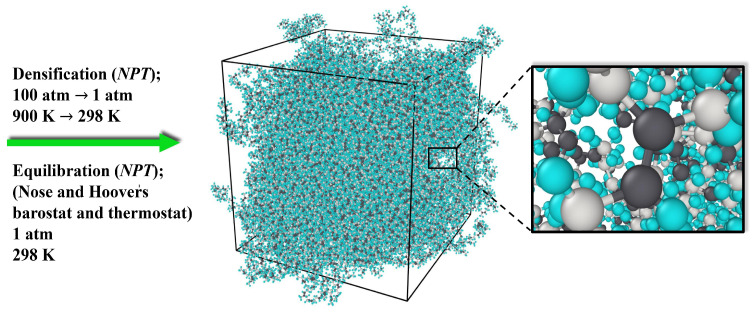
Molecular model of cis-1,4-polyisoprene after densification and equilibration by Nose and Hoover’s barostat and thermostat.

After the distribution of the cis-1,4-polyisoprene chains in the periodic supercell and applying the *NVE* Langevin thermostat, as displayed in Figure 3, the densification process is required. As demonstrated in Figure 4, the *NPT* ensemble was used to gradually compress the polymeric model structure from high temperatures beginning at 900 K and high pressures of 100 atm to the temperature of 293.15 K and a normal pressure of 1 atm. This process was performed once, for 250 ps, at a time step of 0.2 fs for the all-atom model, and it was carried out for 5000 ps at a time step of 0.5 fs for the united-atom model.

To achieve a realistic structure and an equilibrated density at a specific temperature and pressure, the subsequent step is to apply Nose and Hoover’s barostat and thermostat [81,82]. For the all-atom model, damping parameters of 20 and 200 time steps were considered, respectively, for the thermostat and barostat. On the other hand, for the united-atom model, damping parameters of 50 and 500 time steps were applied for the thermostat and barostat. The polymeric model system was equilibrated in an *NPT* ensemble at a temperature of 293.15 K and a pressure of 1 atm, using a time step of 0.2 fs for 300 ps for the all-atom model and a time step of 0.5 fs for 12,000 ps for the united-atom model.

At this point, the system is adequately prepared to achieve the glass transition temperature; consequently, through examining a wide range of temperatures in the *NPT* ensemble at a normal pressure of 1 atm, the relation between the density of the structure and temperatures can be determined.

The normalized heat flux autocorrelation function has to be calculated in order to determine the thermal conductivity. This can be performed by applying sequential ensembles, including *NVT* and *NVE*. Consequently, the energy of the polymeric model structure will be stabilized following the equilibration of the density in step 6 of Figure 1. Therefore, the system is simulated in an *NVT* ensemble at a particular temperature with a simulation time of 2000 ps and a time step of 0.5 fs for the united-atom model. To determine the heat flux autocorrelation function, an *NVE* ensemble is used. At each time step, the heat flux in each direction is then calculated as three components. The Green–Kubo equation is applied to determine the thermal conductivity for each correlation interval [63]. For the united-atom model, the *NVE* ensemble is used with a correlation length of 12 ps and a simulation time of 1200 ps.

**Figure 5:**
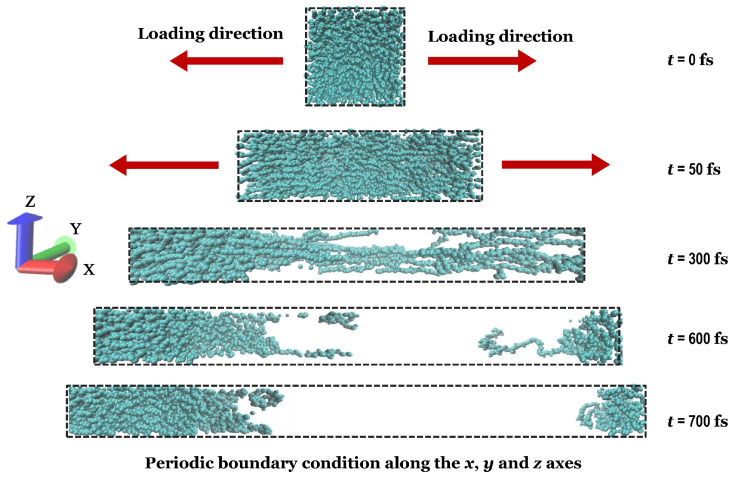
Uniaxial tensile deformation in the x-axis direction at a constant strain rate by applying periodic boundary conditions in three dimensions.

Then, a uniaxial tensile deformation was carried out by inserting the equilibrated structure, which was obtained from step 6 of Figure 1, into the MD code. The deformation simulation was run in LAMMPS by gradually increasing the distance between two opposing sides of the simulation box [10], as illustrated in Figure 5. The uniaxial tensile strain rate is the expanding rate, and its impact on the mechanical characteristics of the polymeric structure will be examined. The strain rate that has been considered in our following investigations is in the magnitude order of (×10^−5^ (1/fs)), based on Chen et al.’s [10] studies. Moreover, comparable strain rates have also been adopted in investigations [83,84,85,86,87] with molecular dynamic simulations. Moreover, the influence of various temperatures on the mechanical properties of the polymer is studied. It should be mentioned that an *NPT* ensemble was used during the tensile test to regulate the temperature of the simulated system at the precise temperature that the structure is exposed to.

### 3.2. Thermal Properties of Cis-1,4-Polyisoprene with United-Atom Force Field in MD Simulations

In this section, thermal properties of a cis-1,4-polyisoprene structure, including thermal conductivity and glass transition temperature at a constant pressure of 1 atm, were investigated in a wide range of temperatures. Each cis-1,4-polyisoprene chain has a certain degree of polymerization of 50 monomers and 250 united atoms (652 atoms) in the united-atom model. The whole produced polymeric system is also composed of 50 cis-1,4-polyisoprene chains and 12,500 united atoms (31,250 atoms).

**Figure 6:**
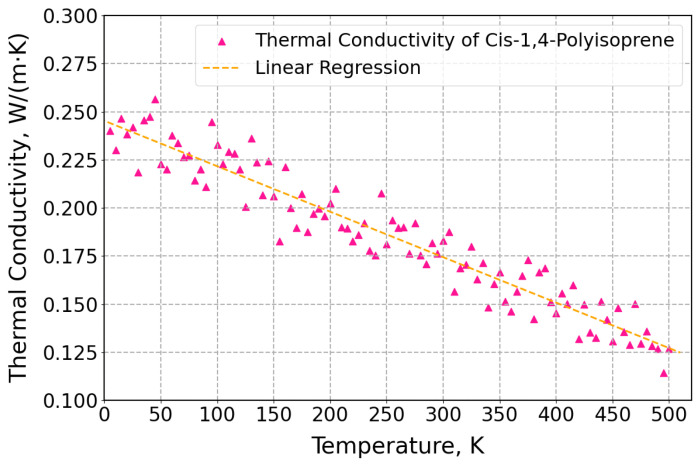
Thermal conductivities for cis-1,4-polyisoprene at a normal pressure of 1 atm.

Thermal conductivities of cis-1,4-polyisoprene for different temperatures from 5 K to 500 K with an interval of 5 K are displayed in Figure 6.

This figure indicates that as the temperature rises, the thermal conductivities of the polymeric structure decrease. This trend is consistent with the experimental findings reported for measurements obtained [88] via the transient hot wire method, which included a temperature range between 260 K and 330 K. Moreover, the Debye temperature for commodity polymers is approximately 180–220 K, which is significantly lower than the typical experimental temperature of 300 K. This means that at lower temperatures, the vibrational modes of the polymer chains are more effectively excited, leading to better thermal transport properties, which in turn results in higher thermal conductivity at lower temperatures [89]. Additionally, in polymers, energy is primarily transferred through bonded connections. At lower temperatures, the energy transfer mechanisms are more efficient, allowing for higher thermal conductivity. Moreover, at higher temperatures, energy transfer through non-bonded contacts becomes more prominent [89]. The contribution of bonded and non-bonded interactions to the thermal conductivity of polyisoprene was studied in [90]. Polymer thermal conductivity is primarily caused by the phonon contribution, which is influenced by molecular structure, chain flexibility, and intermolecular interactions. The thermal conductivity decreases at higher temperatures as a result of enhanced phonon scattering and attenuation brought on by the intensified thermal vibrations of atoms and molecules. Additionally, the polymer will expand thermally at the higher temperature, which contributes to reducing density and increasing free volume, both of which reduce thermal conductivity [91]. The linear regression line is applied to anticipate the acquired thermal conductivity at each temperature.

**Figure 7:**
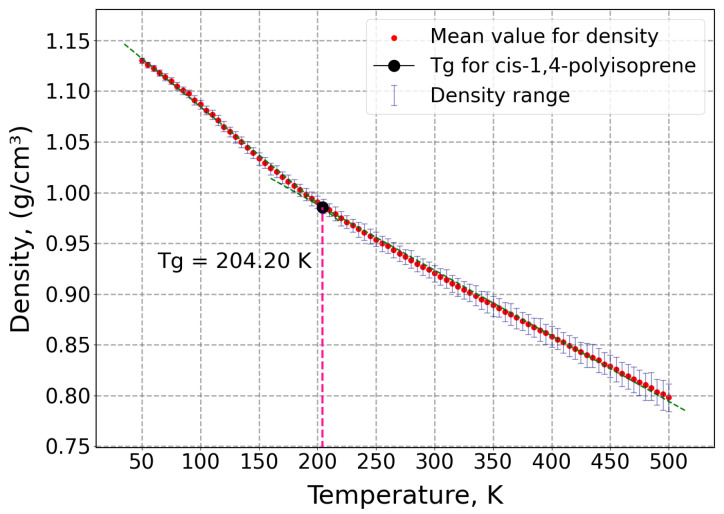
Glass transition temperature *T*_g_ of cis-1,4-polyisoprene at normal pressure of 1 atm for a broad range of temperatures.

In addition, as it was displayed in Figure 7, the glass transition temperature of the polymer was obtained by calculating density for various temperatures from 50 K to 500 K in 5 K intervals under a constant pressure of 1 atm.

As shown in Figure 7, the obtained glass transition temperature of cis-1,4-polyisoprene by MD simulation is 204.20 K, which is in full agreement with the obtained glass transition temperature of 202.15 K in experimental investigations that have been studied by Makhiyanov et al. [92] using the differential scanning calorimetry (DSC) method.

### 3.3. Results and Analysis of Cis-1,4-Polyisoprene Tensile Simulation

#### 3.3.1. Tensile Test of Cis-1,4-Polyisoprene with All-Atom Force Field in MD Simulations

**Figure 8:**
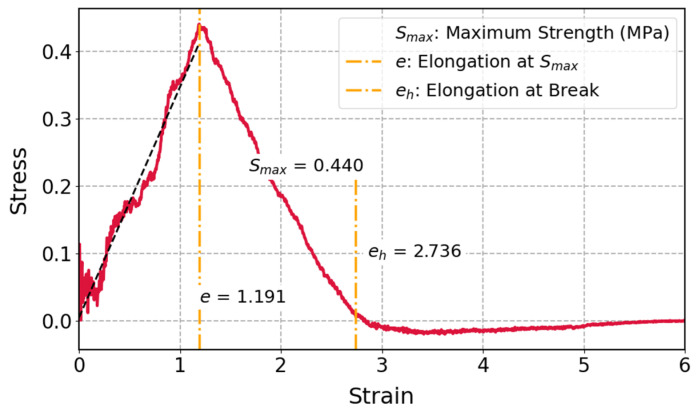
Stress–strain curve of cis-1,4-polyisoprene at 298 K and a strain rate of 1.5 × 10^−5^ (1/fs) with an all-atom force field.

The stress–strain curve of cis-1,4-polyisoprene with an all-atom force field shown in Figure 8 was studied at a temperature of 298 K and a constant strain rate of 1.5 × 10^−5^ (1/fs) and it is clear that the polymeric structure initially illustrates linear elastic behavior. The tensile stress starts declining as it approaches the yield point. When the material ultimately reaches the failure stage, stress keeps decreasing until fracture happens.

The stress–strain response of cis-1,4-polyisoprene, which is displayed in Figure 8, is consistent with the findings of the uniaxial tensile test involving an all-atom force field in Chen et al.’s [10] studies. It has been demonstrated that the polymeric structure shows particular mechanical reactions, which include linear elasticity, yielding, plastic deformation, and ultimate fracture, upon uniaxial tensile deformation. As can be observed in Figure 8, the stress decreases after elastic deformation. A study on the mechanical behaviors of polyisoprene under uniaxial tension for all-atom force field discussed [10] the role of non-bonded potential energy in stress changes in this range. During the uniaxial tensile process, the non-bonded potential energy plays a dominant role in the total system potential energy changes. After reaching the yield point, the non-bonded potential energy begins to decrease. This corresponds to the onset of plastic deformation, where molecular rearrangements, such as chain sliding and disentanglement, occur. The reduction in non-bonded potential energy reflects a weakening of inter-chain interactions, which contributes to the observed drop in stress. Moreover, Zhao et al. [93] mentioned that after reaching the elastic limit, the stress may decrease due to the material’s inability to sustain the same level of stress as it transitions into plastic deformation. This transition is characterized by the rearrangement of atomic structures, which can lead to a reduction in the effective load-bearing capacity of the material. As the bonds start to break or yield, the stress experienced by the material decreases [93,94]. The molecular structure, strain rate, and temperature all have considerable influence on mechanical characteristics. The following sections provide further details on the latter two factors and present an extensive analysis of how they affect the mechanical characteristics of the polymeric structure.

In the applied LAMMPS simulations for the uniaxial tensile test, stress is calculated based on the instantaneous cross-sectional area of the simulation box. Since the system undergoes uniaxial tensile deformation along the x-axis, the cross-sectional area corresponds to the plane perpendicular to this direction, which is defined by the box dimensions in the y- and z-directions. Specifically, we use the product of the instantaneous box lengths in these two directions, denoted as $L_{y}$ and $L_{z}$, respectively. As the material deforms, lateral contraction (Poisson effect) causes $L_{y}$ and $L_{z}$ to change over time, meaning the cross-sectional area dynamically evolves throughout the simulation. This ensures that the computed stress values accurately reflect the system’s real-time geometry during deformation.

Furthermore, there is a need to select a proper force field for MD simulations in order to create a balance between computational cost and molecular detail resolution. United-atom (UA) force fields combine nonpolar hydrogen atoms with their bonded carbon atoms to form single particles, providing a streamlined method. Using united-atom force fields can provide a context in which larger polymeric structures and longer timescales will be simulated by applying constrained computational resources. The united-atom force field is used in simulations in which hydrogen interactions are less of a priority and the bulk characteristics or molecular vibrations are the main priority. Therefore, the united-atom force field was utilized entirely in all of the following simulations to make use of the computational benefits.

#### 3.3.2. Influence of Temperature on Mechanical Properties of Cis-1,4-Polyisoprene with Constant Strain Rate

In this part, the cis-1,4-polyisoprene structure composed of 50 chains is examined under uniaxial tensile deformation with the united-atom force field by applying molecular dynamics (MD) simulations at the pressure of 1 atm. The influence of temperature variations on the mechanical properties of cis-1,4-polyisoprene, including the elasticity modulus (E), maximum strength (S_max_), elongation at maximum strength (e), and elongation at break ($e_{h}$), will be studied.

**Figure 9:**
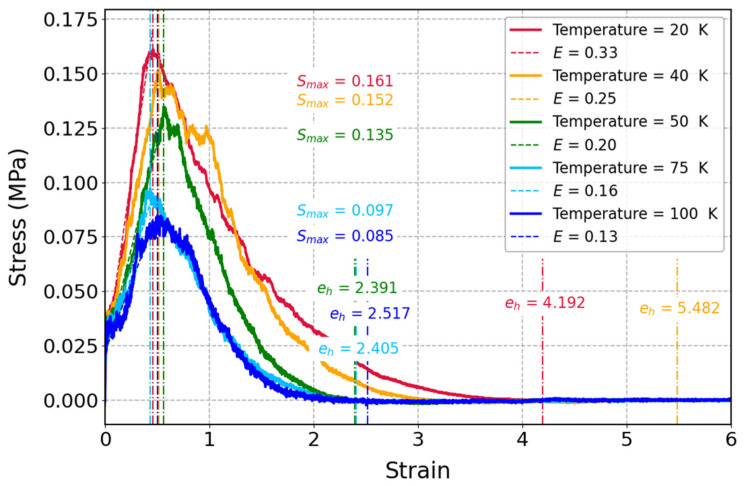
Stress–strain curves of cis-1,4-polyisoprene: influence of temperature on mechanical properties of the polymeric structure composed of 50 chains at a constant strain rate of 3 × 10^−5^ (1/fs) and normal pressure of 1 atm with a united-atom force field.

As it is displayed in Figure 9, mechanical properties of cis-1,4-polyisoprene at a constant strain rate of 3 × 10^−5^ (1/fs) and different temperatures were studied through stress–strain curves. For lower temperatures, the initial gradients of the stress–strain curves are sharper, which indicates a higher elasticity modulus. Based on this observation, the polymeric structure demonstrates higher stiffness under cooler conditions. Moreover, there is an apparent decline in the maximum strength (e), which is in line with the behavior of polymers by increasing temperature. Thermal vibrations of molecules will decrease the intermolecular forces at higher temperatures, which in turn results in decreasing the entire resistance of the material to deformation and facilitating movement of molecular chains.

**Figure 10:**
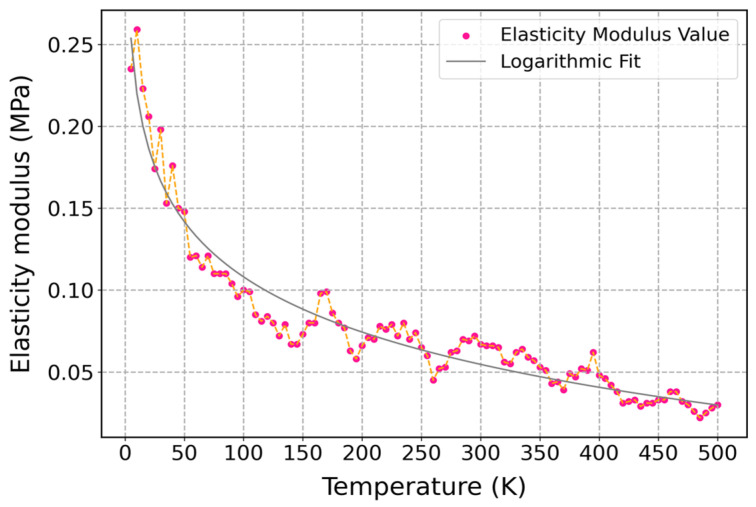
Influence of temperature on the elasticity modulus of the cis-1,4-polyisoprene structure with the united-atom force field at a constant strain rate of 3 × 10^−5^ (1/fs) and normal pressure of 1 atm.

The elastic modulus of cis-1,4-polyisoprene as a function of temperature is illustrated in Figure 10, where the strain rate and pressure are kept constant at 3 × 10^−5^ (1/fs) and 1 atm, respectively. When temperature increases, the elastic modulus of the material decreases. The reduction in the elasticity modulus is an indication of a decline in the stiffness of the material at higher temperatures. Molecular mobility of the polymeric chains at higher temperatures is the main reason for reducing the elasticity modulus at higher temperatures because the kinetic energy of the polymer matrix will increase by increasing temperature, which results in enhancing the segmental motion of polymer chains. This phenomenon can lead to a decrease in intermolecular interactions like van der Waals forces and a decrease in resistance to deformation.

The elastic modulus of cis-1,4-polyisoprene significantly depends on the temperature, especially on the glass transition temperature (*T*_g_), which is around 204 K. The polymer has a high elastic modulus and considerable stiffness below *T*_g_, given that it is in a glassy state with limited molecular mobility. The polymeric structure undergoes a transition from the glassy state to the rubbery state as the temperature exceeds *T*_g_. In the rubbery state, increasing molecular motion of the material results in decreasing the elastic modulus, which shows that the material has less stiffness.

For instance, based on the studies on cis-polyisoprene and trans-polyisoprene blends, the storage modulus decreases by enhancing temperature, while a significant change around *T*_g_ occurs [95]. Moreover, investigation on the bulk modulus of polyisoprene shows similar behavior in which the bulk modulus of the material decreases with increasing temperature, while there is a notable reduction around *T*_g_ [96]. These studies are in agreement with the obtained results in Figure 10, where temperature influences mechanical characteristics, and this behavior is in agreement with the concept of viscoelastic materials. The polymeric structure is in a glassy and brittle state below *T*_g_ with a high elastic modulus, which implies that the material has higher stiffness. However, the material represents rubbery and flexible properties above *T*_g_, and it has a low elastic modulus with less stiffness.

Furthermore, the Williams–Landel–Ferry (WLF) equation [97] implies how viscoelastic characteristics change with temperature, which demonstrates that an increase in temperature corresponds to a logarithmic decrease in the time required to apply equal strain under a constant stress.

**Figure 11:**
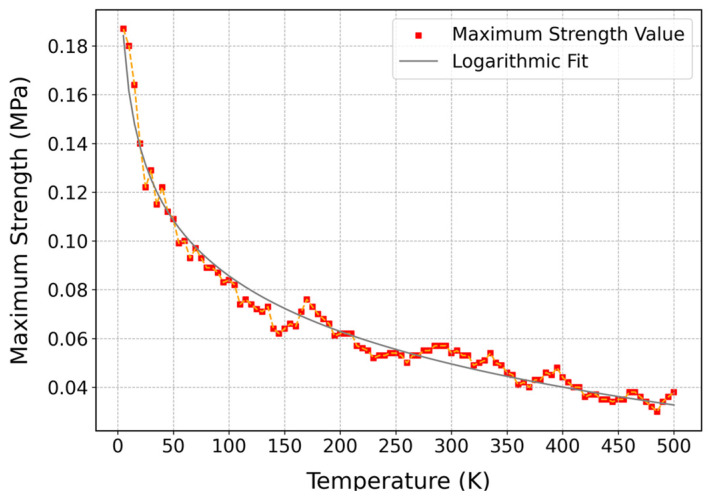
Influence of temperature on maximum strength of cis-1,4-polyisoprene structure with the united-atom force field at a constant strain rate of 3 × 10^−5^ (1/fs) and normal pressure of 1 atm.

As can be observed in Figure 11, the influence of the temperature on the maximum strength of cis-1,4-polyisoprene is illustrated at a constant strain rate of 3 × 10^−5^ (1/fs) and normal pressure of 1 atm. A logarithmic decrease in the maximum strength of cis-1,4-polyisoprene with increasing temperature can be observed in this figure, which can be associated with some interconnected factors. By increasing the temperature, the thermal energy of the polymeric structure is increasing, which results in enhanced molecular motion and reduced intermolecular forces such as van der Waals interactions. This thermal softening brings about a decline in the mechanical strength and stiffness of the material.

Moreover, increasing temperature improves the segmental dynamics of polymer chains, which leads to conformational transitions and allows material to deform more easily. Furthermore, at higher temperatures, thermal expansion increases the free volume of the polymeric structure, which creates a context for polymer chains to move more freely and reduce the density of the polymeric structure, which in turn contributes to a softer material with lower strength. These combinations of factors [98], including thermal softening, enhanced segmental dynamics, and thermal expansion, indicate the inverse relationship between temperature and mechanical strength in cis-1,4-polyisoprene.

**Figure 12:**
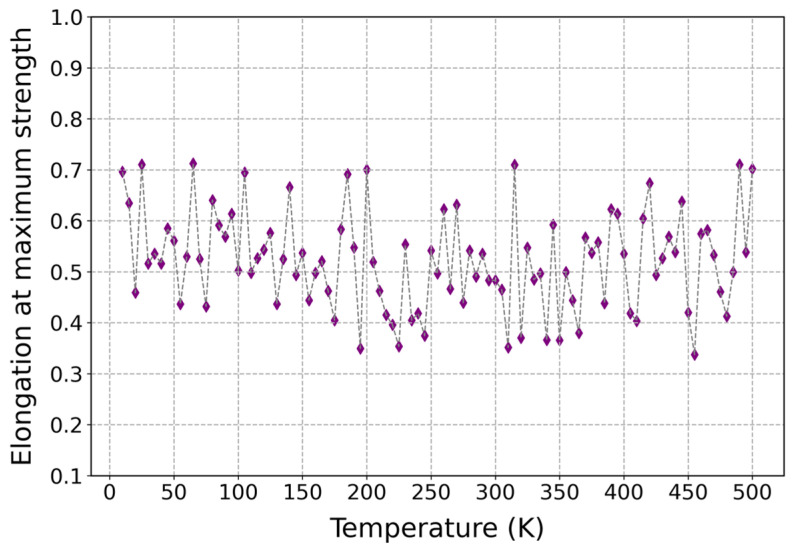
Influence of temperature on the elongation at maximum strength of cis-1,4-polyisoprene structure with the united-atom force field at a constant strain rate of 3 × 10^−5^ (1/fs) and normal pressure of 1 atm.

As can be observed in Figure 12, the influence of temperature on the elongation at maximum strength of cis-1,4-polyisoprene was illustrated at a constant strain rate of 3 × 10^−5^ (1/fs) and normal pressure of 1 atm. According to this figure, the elongation at maximum strength remains nearly constant by increasing temperature, which can be attributed to some factors, including viscoelastic behavior and strain-induced crystallization of cis-1,4-polyisoprene. According to the chemical structure of cis-1,4-polyisoprene, it has viscoelastic behavior and both viscous and elastic features [99]. The balance between viscous and elastic features enables the polymeric structure to stretch and recover without major changes in elongation at maximum strength by increasing temperature. Moreover, the polymeric structure can endure strain-induced crystallization at higher temperatures, which results in enhancing the strength of the material and constraining elongation. Therefore, this fact leads to consistent elongation at maximum strength, irrespective of temperature variations.

**Figure 13:**
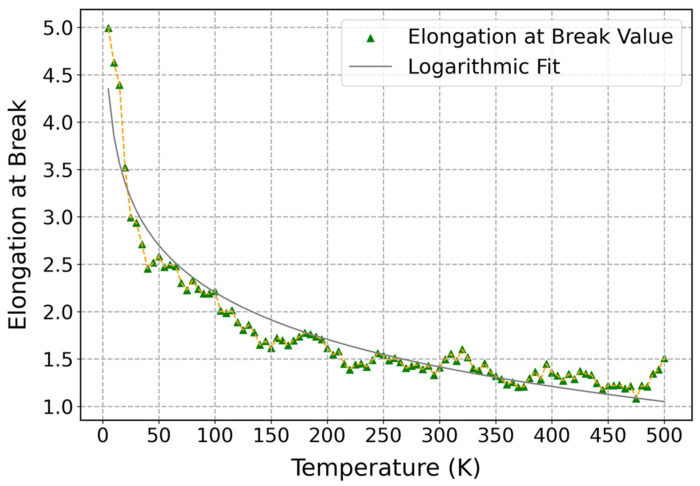
Influence of temperature on elongation at break of cis-1,4-polyisoprene structure with a united-atom force field at a constant strain rate of 3 × 10^−5^ (1/fs) and normal pressure of 1 atm.

Figure 13 shows the influence of temperature on the elongation at break of cis-1,4-polyisoprene, which was displayed at a constant strain rate of 3 × 10^−5^ (1/fs) and normal pressure of 1 atm. According to this figure, a logarithmic decrease in the elongation at break is observed by increasing temperature, which can be attributed to some factors, including thermal softening, viscoelastic behavior, molecular mobility, and stress-aided thermally activated processes [100]. After increasing the temperature, the kinetic energy of the structure increases, and particles will move more freely. This results in decreasing the intermolecular forces that are holding the chains together. Therefore, it leads to a decrease in the stiffness of the polymeric structure and, consequently, the elongation at break of the material. Moreover, due to the viscoelastic nature of polymers, the viscous behavior of polymers becomes more pronounced, making the material deform more easily; consequently, the elongation at break will be decreased. Moreover, higher temperatures can result in decreasing elongation at break by providing necessary energy to break bonds.

#### 3.3.3. Influence of Strain Rate on Mechanical Properties of Cis-1,4-Polyisoprene with Constant Temperature

In this part, the cis-1,4-polyisoprene structure composed of 50 chains is studied under uniaxial tensile deformation with the united-atom force field by applying molecular dynamics (MD) simulations at a constant temperature of 295 K and normal pressure of 1 atm. The influence of strain rate variations on the mechanical properties of cis-1,4-polyisoprene, including the elasticity modulus (E), maximum strength (S_max_), elongation at maximum strength (e), and elongation at break ($e_{h}$), is studied.

**Figure 14:**
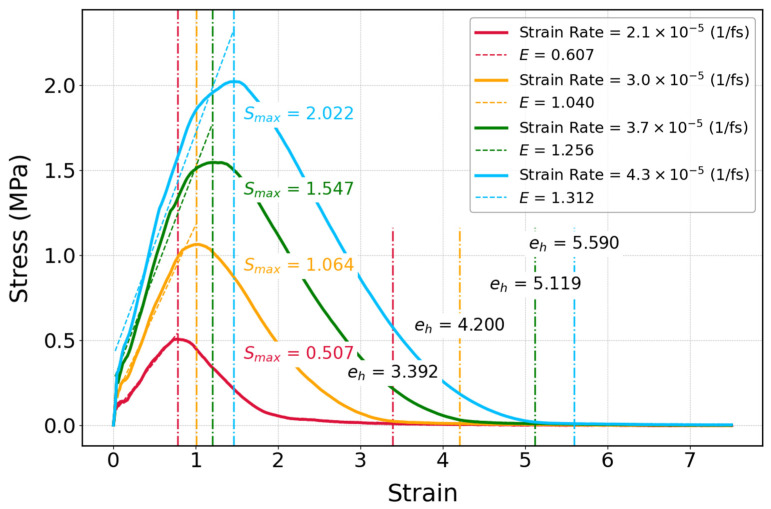
Stress–strain curve of cis-1,4-polyisoprene: influence of strain rate on mechanical properties of the polymeric structure composed of 50 chains at a constant temperature of 295 K and normal pressure of 1 atm with the united-atom force field.

As can be seen in Figure 14, stress–strain curves of cis-1,4-polyisoprene at different strain rates are illustrated in order to examine the mechanical properties of the material. According to the stress–strain curves of cis-1,4-polyisoprene, the initial slopes of the curves are less steep at lower strain rates, which implies a lower elasticity modulus. This particular trend shows that the material has higher stiffness at higher strain rates. There is also an increasing trend in maximum strength (S_max_), elongation at maximum strength (e), and elongation at break ($e_{h}$) by enhancing strain rate.

The correlation between strain rate and mechanical properties of polymers is attributed to the time-dependent viscoelastic nature of polymers from a molecular dynamic point of view. The polymer chains do not have enough time in order to be reorganized and relaxed under deformation at higher strain rates, which leads to the enhancement of resistance to deformation and higher elasticity modulus and stiffness. In contrast, polymer chains have more time to realign and relieve stress at lower strain rates, which results in lower stiffness of the material. Therefore, mechanical properties of viscoelastic materials are influenced strongly by the applied strain rate.

Candau et al. [101] carried out an experiment on synthetic polyisoprene by wide-angle X-ray scattering; they discovered that strain-induced crystallization will occur at larger stretching ratios during low strain rate stretching. It implies that polyisoprene displays delayed strain-induced crystallization at lower strain rates, which correlates with the decline in the stiffness of the material. Furthermore, another investigation on the influence of strain rate on the mechanical properties of polymeric structures shows that elasticity moduli and yield stresses will be increased by enhancing strain rate [102]. Therefore, this general trend in polymers is in agreement with the obtained results related to cis-1,4-polyisoprene, as shown in Figure 14.

**Figure 15:**
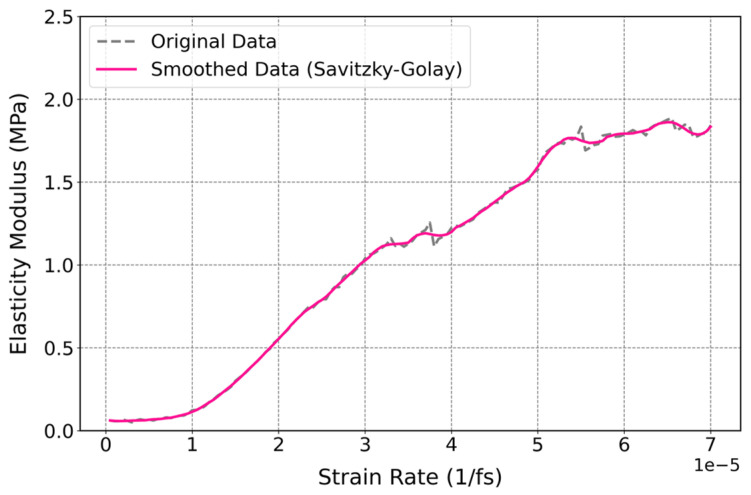
Influence of strain rate on the elasticity modulus of the cis-1,4-polyisoprene structure with a united-atom force field at a constant temperature of 295 K and normal pressure of 1 atm.

The elastic modulus of cis-1,4-polyisoprene as a function of temperature is illustrated at a constant temperature of 295 K and normal pressure of 1 atm in Figure 15. The elastic modulus increases by raising the strain rate, which represents an increase in the stiffness of the material. According to experimental investigations of Berto et al. [103], they obtained mechanical properties of polyisoprene through two methods, including tensile test analysis and dynamic mechanical analysis (DMA). The elastic modulus obtained from tensile tests for polyisoprene is 1.41 ± 0.11 MPa, with an elongation at break of 600 ± 86% at 298 K. These experimental results are in agreement with the elastic modulus of 1.31 MPa and elongation at break of 560% observed in the current simulations at 295 K and at the strain rate of 4.3 × 10^−5^ (1/fs) displayed in Figure 14.

Figure 15 illustrates that the elasticity modulus of the cis-1,4-polyisoprene structure becomes almost constant at higher strain rates, which is mainly a result of saturation in the mechanical response of the polymer. A study reports an approximate elasticity modulus value around 2 MPa for cis-1,4-polyisoprene crosslinked with sulfur [104]. Additionally, it can be inferred from the work of Akinay et al. [105] that the elasticity modulus of untreated or non-crosslinked polyisoprene would be lower than that of the crosslinked structure. This aligns with our current findings, where the elasticity modulus of the untreated polyisoprene is observed to be below 2 MPa.

The increase in elastic modulus by increasing strain rate is as a result of the viscoelastic behavior of polymers. The viscoelastic characteristics of polymeric structures become more prominent for higher strain rates [106]. The increase in elastic modulus by increasing strain rate can be as a result of the balance between elastic and viscous responses in the polymeric structure. At lower strain rates, the polymer chains can slide and rearrange relative to each other, which results in transferring energy through relaxation processes. Therefore, the increase in molecular mobility causes a lower modulus as the viscous nature of the material dominates. Contrary to this, at higher strain rates, chain mobility is noticeably reduced and limited, which in turn results in the suppression of the relaxation process. Thus, a primarily elastic response would be observed in polymeric structure due to the increase in the elasticity modulus.

**Figure 16:**
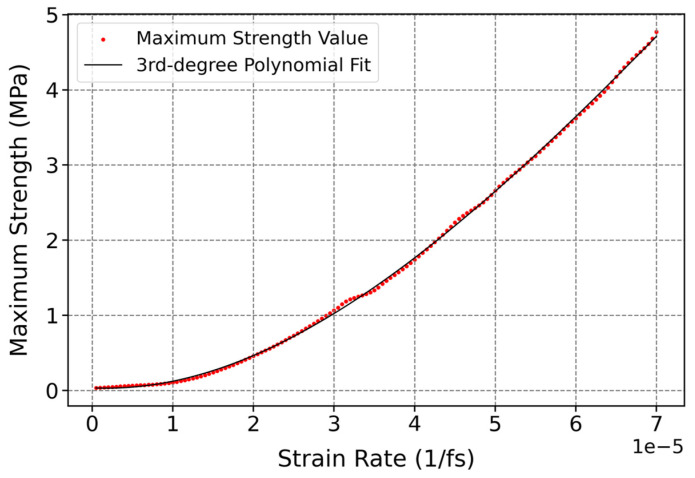
Influence of strain rate on maximum strength of cis-1,4-polyisoprene structure with the united-atom force field at a constant temperature of 295 K and normal pressure of 1 atm.

As can be seen in Figure 16, the influence of strain rate on the maximum strength of cis-1,4-polyisoprene was demonstrated at a constant temperature of 295 K and normal pressure of 1 atm. A polynomial increase in the maximum strength of cis-1,4-polyisoprene by increasing strain rate can be observed in this figure, which can be associated with some interconnected factors. Since polymers have both viscous and elastic properties, the elastic reaction of the polymer at a higher rate of deformation becomes more prominent, which contributes to increasing strength [107]. In addition, polymer chains are more capable of becoming aligned and oriented under high strain rates, which in turn results in increasing the load-bearing capacity of the material. Furthermore, strain-induced crystallization takes place by increasing the rate of deformation, which contributes to increasing crystallization and, consequently, the maximum strength of the polymer [101].

**Figure 17:**
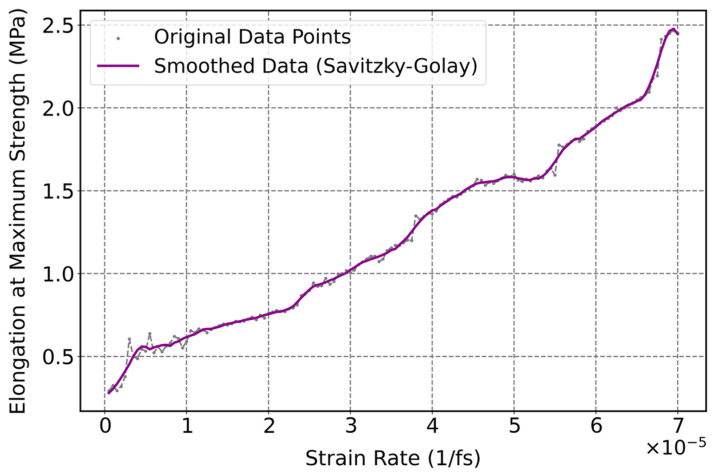
Influence of strain rate on elongation at maximum strength of the cis-1,4-polyisoprene structure with the united-atom force field at a constant temperature of 295 K and normal pressure of 1 atm.

Figure 17 illustrates the effect of strain rate on the elongation at maximum strength of cis-1,4-polyisoprene at a constant temperature of 295 K and normal pressure of 1 atm. This figure displays an increase in the elongation at maximum strength by increasing strain rate, which can be due to a number of interrelated factors. Polymer chains have insufficient time to relax and rearrange at a higher rate of deformation, which results in temporary stiffness of the material and the ability to withstand higher elongation before reaching stress peaks. Moreover, under rapid deformation, polymer chains would be restricted from disentangling and sliding past each other so that the deformation takes place over a larger strain range before reaching maximum strength. Furthermore, at higher strain rates, polymer matrices store greater mechanical energy [10], which enhances the resistance of the material to rupture and elongation at maximum strength.

**Figure 18:**
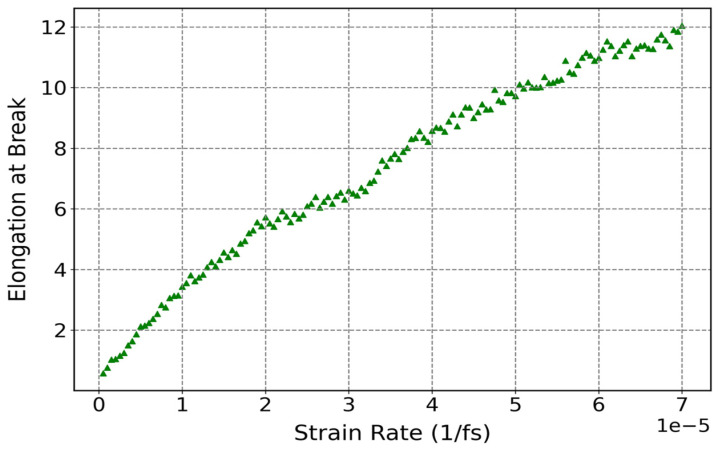
Influence of strain rate on elongation at break of cis-1,4-polyisoprene structure with united-atom force field at a constant temperature of 295 K and normal pressure of 1 atm.

Figure 18 illustrates how strain rate affected the elongation at break of cis-1,4-polyisoprene at a constant temperature of 295 K and normal pressure of 1 atm. According to this figure, the elongation at break of cis-1,4-polyisoprene rises as the strain rate increases. This phenomenon can be attributed to a number of interrelated factors that are highlighted in Figure 17, including insufficient relaxation time, restriction of polymer chain disentanglement and sliding, and storage of mechanical energy in the polymer matrix.

## 4. Conclusions

In this study, thermal properties of cis-1,4-polyisoprene, including thermal conductivity and glass transition temperature (*T*_g_), were studied analytically with a united-atom force field model by molecular dynamics (MD) simulations. According to the obtained results, the thermal conductivity of cis-1,4-polyisoprene is noticeably dependent on temperature so that it decreases with increasing temperature as a result of enhanced phonon scattering and energy transfer mechanism. The glass transition temperature of cis-1,4-polyisoprene was also obtained at 204.20 K, which represents a temperature at which the amorphous polymeric structure shifts from a hard, glassy state to a soft, rubbery state.

In order to investigate the influence of temperature on the mechanical properties of cis-1,4-polyisoprene, the uniaxial tensile test was studied at a constant pressure of 1 atm and strain rate of 3 × 10^−5^ (1/fs) by considering the united-atom force field model by using MD simulation. A logarithmic decrease in the elastic modulus, maximum strength, and elongation at break of the material will occur by increasing temperature. The reduction in the elasticity modulus is an indication of a decline in the stiffness of the material at higher temperatures. A notable reduction in elasticity modulus has occurred at 204 K, which shows the influence of glass transition temperature directly on the mechanical properties of the material. Moreover, the elongation at maximum strength remains nearly constant by increasing temperature, which attributes to the thermal stability of the material, viscoelastic behavior, and strain-induced crystallization of the material in this state.

In addition, the influence of strain rate on the mechanical properties of cis-1,4-polyisoprene with a united-atom force field model was studied at a constant temperature of 295 K and pressure of 1 atm. The elasticity modulus of the structure increases by enhancing the strain rate due to the viscoelastic behavior of the structure. The elasticity modulus becomes almost constant at 1.7 MPa, which is because of the saturation in the mechanical response of the polymer at higher strain rates. This is comparatively in line with the fact that the elasticity modulus of the untreated polyisoprene is below 2 MPa. The maximum strength of the structure increases by enhancing the rate of deformation, which is on account of increasing load-bearing capacity and strain-induced crystallization of the material. The elongation at maximum strength and elongation at break will also increase by enhancing the rate of deformation, which would be attributed to insufficient relaxation time, restriction of polymer chain disentanglement, and storage of mechanical energy in the polymer matrix. These simulation-based predictions provide valuable guidance for experimentalists, which is significantly lowering the time and cost associated with laboratory initiatives. They facilitate the development of materials that are designed for specific applications across various sectors, including aerospace, automotive, and industrial engineering.

## Figures and Tables

**Table 1:** All-atom force field parameters utilized for MD simulations. The typical factor of ½ in stretching and bending interactions is considered in ***k*_a_** and ***k*_b_** [78].

	Force Field Parameters for cis-1,4-Polyisoprene in All-Atom Model			
**Atom Type**	$U_{LJ}=4\epsilon \left[{\left(\frac{\sigma }{r}\right)}^{12}-{\left(\frac{\sigma }{r}\right)}^{6}\right]$	$\epsilon \;\left[\frac{kcal}{mol}\right]$	$\sigma \;\left[Å\right]$	
81, 81	$-{CH}_{2}-,{-CH}_{2}-$	0.066	3.5	
80, 80	${CH}_{3}-,{CH}_{3}-$	0.066	3.5	
85, 85	H−C,H−C	0.030	2.5	
86, 86	$=C\left(-C\right)-C,=C\left(-C\right)-C$	0.076	3.55	
87, 87	$=C\left(-H\right)-C,=C\left(-H\right)-C$	0.076	3.55	
89, 89	H−C,H−C	0.03	2.42	
80, 89	${CH}_{3}-,H-C$	0.044	2.910	
81, 85	$-{CH}_{2}-,H-C$	0.044	2.910	
81, 86	$-{CH}_{2}-,=C\left(-C\right)-C$	0.071	3.525	
81, 87	$-{CH}_{2}-,=C\left(-H\right)-C$	0.071	3.525	
81, 89	$-{CH}_{2}-,H-C$	0.044	2.910	
85, 86	$H-C,=C\left(-C\right)-C$	0.048	2.979	
85, 87	$H-C,=C\left(-H\right)-C$	0.048	2.979	
85, 89	H−C,H−C,	0.030	2.460	
86, 87	$=C\left(-C\right)-C,=C\left(-H\right)-C$	0.076	3.550	
86, 89	$=C\left(-C\right)-C,H-C$	0.048	2.931	
87, 89	$=C\left(-H\right)-C,H-C$	0.048	2.979	
	$U_{bond}=k_{b}\;{\left(r-r_{0}\right)}^{2}$	$k_{b}\;\left[\frac{kcal}{mol\;{Å}^{2}}\right]$	$r_{0}\;\left[Å\right]$	
81, 81	$-{CH}_{2}-,{-CH}_{2}-$	268.0	1.524	
80, 85	${CH}_{3}-,H-C$	340.0	1.09	
81, 86	$-{CH}_{2}-,=C\left(-C\right)-C$	317.0	1.51	
87, 89	$=C\left(-H\right)-C,H-C$	340.0	1.08	
86, 87	$=C\left(-C\right)-C,=C\left(-H\right)-C$	549.0	1.34	
	$U_{angle}=k_{a}\;{\left(\theta -\theta_{0}\right)}^{2}$	$k_{a}\;\left[\frac{kcal}{mol\;{rad}^{2}}\right]$	$\theta_{0}\;\left[degrees\right]$	
85, 81, 85	$H-C,{-CH}_{2}-,H-C$	33.0	107.8	
81, 81, 85	${-CH}_{2}-,{-CH}_{2}-,H-C,$	37.5	110.7	
85, 81, 86	$H-C,{-CH}_{2}-,=C\left(-C\right)-C$	35.0	109.5	
81, 81, 87	${-CH}_{2}-,{-CH}_{2}-,=C\left(-H\right)-C$	63.0	111.1	
80, 86, 81	${CH}_{3}-,=C\left(-C\right)-C,{-CH}_{2}-$	70.0	130.0	
81, 87, 89	${-CH}_{2}-,=C\left(-H\right)-C,H-C$	35.0	117.0	
89, 87, 86	$H-C,=C\left(-H\right)-C,=C\left(-C\right)-C$	35.0	120.0	
81, 86, 87	${-CH}_{2}-,=C\left(-C\right)-C,=C\left(-H\right)-C$	70.0	124.0	
85, 80, 85	$H-C,{CH}_{3}-,H-C$	33.0	107.8	
85, 81, 87	$H-C,{-CH}_{2}-,=C\left(-H\right)-C$	35.0	109.5	
81, 87, 86	${-CH}_{2}-,=C\left(-H\right)-C,=C\left(-C\right)-C$	70.0	124.0	
	$U_{diherdal}=\sum_{j=1}^{3}\left(\frac{K_{j}}{2}\left[1+{\left(-1\right)}^{j+1}\;cos\left(j\varphi \right)\right]\right)$	$K_{1}\;\left[\frac{kcal}{mol}\right]$	$K_{2}\;\left[\frac{kcal}{mol}\right]$	$K_{3}\;\left[\frac{kcal}{mol}\right]$
85, 81, 81, 85	$H-C,{-CH}_{2}-,{-CH}_{2}-,H-C$	0.0	0.0	0.3
85, 81, 81, 87	$H-C,{-CH}_{2}-,{-CH}_{2}-,=C\left(-H\right)-C$	0.0	0.0	0.366
81, 81, 87, 89	${-CH}_{2}-,{-CH}_{2}-,=C\left(-H\right)-C,H-C$	0.0	0.0	0.468
81, 81, 86, 80	${-CH}_{2}-,{-CH}_{2}-,=C\left(-C\right)-C,{CH}_{3}-$	2.817	−0.169	0.543
81, 81, 86, 87	${-CH}_{2}-,{-CH}_{2}-,=C\left(-C\right)-C,=C\left(-H\right)-C$	0.346	0.405	−0.904
85, 80, 86, 81	$H-C,{CH}_{3}-,=C\left(-C\right)-C,{-CH}_{2}-$	0.0	0.0	0.3
85, 81, 87, 89	$H-C,=C\left(-H\right)-C,=C\left(-C\right)-C$	0.0	0.0	0.318
85, 81, 86, 87	$H-C,{-CH}_{2}-,=C\left(-C\right)-C,=C\left(-H\right)-C$	0.0	0.0	−0.372
81, 87, 86, 80	${-CH}_{2}-,=C\left(-H\right)-C,=C\left(-C\right)-C,{CH}_{3}-$	0.0	14.0	0.0
81, 86, 87, 89	${-CH}_{2}-,=C\left(-C\right)-C,=C\left(-H\right)-C,H-C$	0.0	14.0	0.0
85, 81, 87, 89	$H-C,{-CH}_{2}-,=C\left(-H\right)-C,H-C$	0.0	0.0	0.318
85, 81, 87, 86	$H-C,{-CH}_{2}-,=C\left(-H\right)-C,=C\left(-C\right)-C$	0.0	0.0	−0.372
	$U_{improper}=k_{improper}\;{\left(x-x_{0}\right)}^{2}$			
85, 80, 86, 87	$H-C,{CH}_{3}-,=C\left(-C\right)-C,=C\left(-H\right)-C$	15.0	180.0	
85, 81, 86, 87	$H-C,{-CH}_{2}-,=C\left(-C\right)-C,=C\left(-H\right)-C$	15.0	180.0	
81, 81, 86, 87	${-CH}_{2}-,{-CH}_{2}-,=C\left(-C\right)-C,=C\left(-H\right)-C$	15.0	180.0	
81, 81, 86, 80	${-CH}_{2}-,{-CH}_{2}-,=C\left(-C\right)-C,{CH}_{3}-$	15.0	180.0	
80, 86, 87, 89	${CH}_{3}-,=C\left(-C\right)-C,=C\left(-H\right)-C,H-C$	15.0	180.0	
81, 86, 87, 89	${-CH}_{2}-,=C\left(-C\right)-C,=C\left(-H\right)-C,H-C$	15.0	180.0	
85, 81, 87, 86	$H-C,{-CH}_{2}-,=C\left(-H\right)-C,=C\left(-C\right)-C$	15.0	180.0	
81, 81, 87, 89	${-CH}_{2}-{,-CH}_{2}-,=C\left(-H\right)-C,H-C$	15.0	180.0	

## Data Availability

Data are contained within the article.
